# Reputation and Responsibility: A Qualitative Investigation of Parents' Experiences of Open Dialogue School Meetings

**DOI:** 10.1111/jmft.70030

**Published:** 2025-05-15

**Authors:** Ben Ong, Andrea McCloughen, Sarah Farrell‐Whelan, Niels Buus

**Affiliations:** ^1^ Monash Nursing and Midwifery, Faculty of Medicine, Nursing and Health Sciences Monash University Melbourne Victoria Australia; ^2^ Susan Wakil School of Nursing and Midwifery, Faculty of Medicine and Health The University of Sydney Sydney New South Wales Australia; ^3^ Wellbeing Coordinator Korowal School NSW Hazelbrook New South Wales Australia; ^4^ Department of Public Health Aarhus University Aarhus Denmark

**Keywords:** collaboration, family therapy, open dialogue, school

## Abstract

Open Dialogue approaches to family therapy emphasize the voicing of multiple perspectives in a supportive collaborative environment. In a novel application of Open Dialogue, this study explored how parents of students at an Australian independent school experienced Open Dialogue meetings within a school setting. Using reflexive thematic analysis, we analyzed 4 h of audio recordings of three focus groups involving 14 parents. Parents reported that Open Dialogue meetings promoted closer relationships between the school, parents, and students and focused on adapting to the needs of the student. However, Open Dialogue meetings also interacted with parents' expectations and the school's reputation for student wellbeing to amplify parents' perceptions of the school's responsibility. Open Dialogue meetings promoted closer collaborative relationships between parents, students, and staff. Clinicians need to be mindful of broader social attitudes that influence a parent's experience, and to set up appropriate expectations to mitigate potential problems.

## 1 Introduction

The early identification, assessment, and treatment of mental health problems is essential to mitigate negative future outcomes (McGorry et al. 2022). The first onset of a mental health disorder occurs in 34.6% of people by the age of 14 years and rises to 48.4% by the age of 18 years (Solmi et al. 2022). International trends suggest that the percentage of young people experiencing psychological distress is increasing at alarming rates (Brennan et al. 2021; Centers for Disease Control and Prevention 2020). As almost half of people experience the onset of mental health disorders before the age of 18 years it is essential for parents, schools, and other supports to be active and engaged in monitoring and supporting young people to avoid future negative outcomes.

Family therapists play an important role through their understanding of systemic relations. This is especially true for young people who are embedded within multiple overlapping systemic structures such as family, school, friends, and broader professional and social supports. Apart from parents and the family, young people spend significant amounts of time at school. Schools therefore play an essential role in monitoring for early warning signs and activating supports, referrals or intervening when students show signs of mental health problems (Evans et al. 2023; Hass and Ardell 2022; Wines et al. 2024). But simply referring young people to external services is no longer enough. Schools are increasingly encouraged to form collaborative relationships with parents to jointly address education and mental health problems (New South Wales Government 2017; Queensland Government 2020) including a sensitivity and respect for the individual needs of students and families (Garner 2020; Wines et al. 2024). This involves schools taking an empathic and family‐centered approach (Evans et al. 2023; Nabors and Bartlett 2024) that actively engages and works with parents so the school can better understand the pressures at home and better support children while they are at school (Garner 2020). The responsibility for the mental health and wellbeing of children thus becomes shared between parents, school staff, and therapists who have professional contact with children. Family therapists working either independently or in schools thus have to navigate a variety of external social pressures on various members of the extended system and attempt to create productive collaborations between multiple stakeholders including school counselors, teachers, parents, external health providers, and the children themselves (Hass and Ardell 2022; Wines et al. 2024).

One approach that combines a client‐centered, multisystemic, and collaborative approach to mental health is Open Dialogue (Brown and Mikes‐Liu 2015). Originally developed in a service for psychosis in rural Finland (Haarakangas et al. 2007), Open Dialogue emphasizes providing immediate help, including the social network, flexibly adapting treatment to the specific needs of the client and family, having multiple and consistent clinicians in each meeting, tolerating uncertainty and being open to different treatment options, and promoting a dialogue between all participants. Therapists thus “follow the lead” of the participants (Ong and Buus 2021, 250). The session begins with an open question such as “what would be the best way to begin?” and focuses on how the participants would like to use the meeting (Olson et al. 2014, 9). Counselors are advised not to seek agreement nor rush to conclusions but instead tolerate uncertainty and continue to promote dialogue and discussion between the various perspectives of the participants (Olson et al. 2014). The principles of tolerating uncertainty and dialogism can result in a slower process where therapists invest longer periods of time in drawing out and understanding the unique perspectives of each person and avoid favouring one particular perspective (Ong and Buus 2021). The aim is for all participants to feel that their positions are heard and taken seriously. Open Dialogue thus focuses on establishing a family‐led dialogical conversation rather than a skills‐based training or intervention approach (Haarakangas et al. 2007; Seikkula and Arnkil 2006). Open Dialogue can provide schools with an alternative approach to meetings as the role of the therapist or counselor is not centered around collecting assessment information and designing interventions but rather in eliciting different perspectives and further dialogues among the participants who collaboratively develop their own solutions (Ong et al. 2021).

In healthcare settings, Open Dialogue meetings are regularly viewed as positive experiences by parents (Buus et al. 2021). Parents have reported that Open Dialogue meetings created a sense of safety in an open and nonhierarchical atmosphere where they felt understood, accepted, and validated (Buus et al. 2021; Sidis et al. 2020) as well as supported and hopeful about the future (Clement and McKenny 2019). However, Open Dialogue meetings can be difficult to organize and fit into the routines of families (Buus and McCloughen 2022), especially if close family members or external professionals cannot or do not want to attend (Florence et al. 2021). Meetings can also be emotionally challenging with discussions of problems that can be very exposing and encompass feelings of failure, embarrassment, and guilt around family dynamics (Buus and McCloughen 2022; Twamley et al. 2021). As therapists focus on facilitating a dialogical conversation and resist claiming expertise and giving advice, more work is required of the family to think about their situation and what changes they need to make (Buus and McCloughen 2022). Some family members prefer more conventional services with clinicians providing diagnoses and expert advice and direction (Buus et al. 2021; Gidugu et al. 2021).

To date the implementation of Open Dialogue has largely been initiated by mental health services, which is then offered to clients accessing that mental health service (Buus et al. 2021). To our knowledge, there is no publicly documented examples, nor research, when Open Dialogue meetings have been widely implemented by a school. This is a novel area for the use of Open Dialogue as school students often represent a nonclinical sample. Open Dialogue in schools has potential to improve student wellbeing by intervening early, including and activating both the professional and personal supports around a young person, actively and sensitively seeking the input from young people and their families on what is important to them, and the ability to streamline communication, problem‐solving, and planning as important people are present in each meeting.

The study site for this study is an Australian school that has independently adopted the use of Open Dialogue in meetings with families when there is an expressed concern about the student. This study focuses on parents' experiences of participating in these meetings including what they view as useful and less useful aspects of the approach. As family therapists are frequently involved with young people and their families, this study provides useful insights on how such a collaborative‐dialogic approach can be implemented outside a strictly mental health setting, and how such an approach is received by parents and what adaptions are necessary.

## 2 Methods

### 2.1 Context

The study site was an independent, coeducational, and secular school with students from kindergarten to year 12. It is a small school with approximately 30 teachers and 250 students, situated in a village locality 100 kms from Sydney (population approx. 5000 people). The area is represented by people who are Australian born and have a higher level of education and income than the state and national averages (Australian Bureau of Statistics 2021).

Before 2022, the school's wellbeing team had trialed various forms of meetings with students and parents. These included more traditional school counselor and parent meetings characterized by the counselor leading the conversation and focusing on assessment of student problems and developing professional‐led interventions (Brigman et al. 2021). In addition, the wellbeing team had been trialing an Open Dialogue approach for some families and students. These meetings were led by the wellbeing staff and included students, parents, teachers, and followed a family and student‐led style of conversation that sought to elicit multiple perspectives and develop collaborative responses to problems. Subsequently, the school management made a commitment to make Open Dialogue an integral part of the school culture and the primary form of meeting when involving parents and students.

Before the commencement of the 2022 academic year, the school management arranged for all staff to receive foundational Open Dialogue training of 5 days for the leadership group and 3 days for teachers. The intention was to prepare teachers to participate in Open Dialogue meetings with the school's wellbeing team. This training was facilitated by the second, third and fourth authors and relied heavily on experiential learning where staff practiced dialogical questioning and reflecting conversations (Ong and Buus 2021). Staff members also practiced being in the “client” position where they were interviewed about an important but not emotionally charged issue in their life. This was to promote greater empathy in staff about the experience of Open Dialogue from client perspective. After the training, meetings with students and families were generally led by wellbeing staff with teachers as active participants along with the student and family member. The wellbeing staff were mental health accredited social workers each with over 20 years of experience working with schools and young people. Two had completed an advanced 3‐year training as an Open dialogue therapist or trainer run by facilitators from Finland.

Open Dialogue meetings were originally initiated by a teacher if they identified any form of student wellbeing or learning issue that could benefit from parental participation. These included things like, attendance problems, attention issues, disruption or withdrawal in class, problems with teacher or peer relationships, academic difficulties or disengagement, adapting to neurodivergence, as well as possible signs of mental health problems. As the meetings became more commonplace, some parents began initiating and requesting Open Dialogue meetings from the school.

### 2.2 Recruitment of Participants

All parents who had experienced at least one Open Dialogue meeting were invited to participate in this study via an email sent by the school's administration coordinator. Interested parents who responded were given more detailed information on the research project and provided written informed consent before the study began. Participants were not financially compensated.

### 2.3 Data Collection

Data were collected in July 2022 via audio recordings of three focus groups totaling 3:56 h of recordings ranging from 1:04 to 1:27 h. We conducted focus groups because they can promote a supportive environment and also encourage spontaneous interactive discussions that develop new ideas between participants (Roller and Lavrakas 2015). Participants attended one of the three focus groups depending on the time of day that they were available. Two facilitators external to the school (the first author and a different colleague) ran the focus groups, which were centered around five statements for discussion (Table 1). These statements focused on the parents' experiences and outcomes of the Open Dialogue meetings as well as contextual issues such as perceptions on the role of teachers in student wellbeing. The facilitators had experience in conducting and researching Open Dialogue but had no prior involvement with the implementation at the school.

**Table 1:** Focus group questions and prompts.

1.	Please share your experiences of participating in an Open Dialogue network meeting at [the school]. What made an impression on you? *Prompt* (if needed): Please explain whether there was clarity around the purpose and structure of the network meeting.
2.	Please explain your agreement/disagreement with the statement: I noticed changes in my child and/or family following network meetings. *Prompt* (if needed): Please describe whether you noticed any effects or outcomes because of the network meetings.
3.	Please explain your agreement/disagreement with the statement: [The school] has a responsibility for my child's mental health and well‐being. *Prompt* (if needed): Please describe your perspective about whether teachers at [The school] should intervene in the psychosocial aspects of your child's life.
4.	Please explain your agreement/disagreement with the statement: The network meetings felt awkward and somewhat unsafe. *Prompt* (if needed): Please provide some broad examples to illustrate any aspects of the network meeting which you did not like or disagreed with. Please share whether you felt the moderators favored certain topics or participants during the meeting.
5.	Please explain your agreement/disagreement with the statement: Unexpected things were said during and after the network meeting. *Prompt* (if needed): Please explain to what extent you felt heard during and after the meeting.

Participants included 14 parents with children in primary school (*n* = 7), high school (*n* = 4), or both primary and high school (*n* = 3). The family structures of participants varied with single or multiple children, mixed and same sex parents, and coupled and single parent families. Participants had been involved in between one to over six Open Dialogue meetings, occurring in the first half of 2022. Participants reported that the composition of meetings varied, but at a minimum included the school counselor, a teacher, and at least one parent. The student was mostly, but not always, present and some meetings also included another parent or family member and other school staff such as a year coordinator, welfare staff, or a member of the leadership team.

### 2.4 Analytic Approach

For this analysis, we adopted a social constructionist epistemology that is consistent with reflexive thematic analysis (Braun and Clarke 2022), a qualitative research method that would support analysis of a range of parental experiences. We followed the phases and quality strategies of reflexive thematic analysis as described by Braun and Clarke (2022). The first author transcribed and checked all focus group transcripts against the original recordings. The first and last author inductively open‐coded the full data set independently. After this initial, open coding the first and last authors discussed their coding choices to add depth to the analysis by developing new ideas, ensuring comprehensive, internally consistent, and coherent coding. With these discussions in mind, the first author completed a second round of coding and constructed codes into initial preliminary themes. These initial themes were discussed with all authors to garner additional perspectives, and promote internal consistency, defensibility, and distinctiveness to the constructed themes. During these discussions, we noticed how some parents introduced issues of responsibility of the school to make changes rather than more collaborative outcomes, which are expected in Open Dialogue theorizing and literature (e.g., Haarakangas et al. 2007). To interpret these unexpected findings, we conducted a broader reading of the literature (Timmermans and Tavory 2022) around parenting culture leading to the concept of “intensive parenting” as a way of reconciling the differences between conventional Open Dialogue theorizing and the study findings.

The entire analytic process was supported through regular reflexive journaling to facilitate deeper reflection on the coding and development of themes, to promote connections between concepts, and to raise awareness and discussions about personal assumptions and responses to the data that may influence the analytic process (Braun and Clarke 2022). The team of authors have a range of experiences in the Open Dialogue approach including clinical, training, and research. While the authors have a philosophical affinity with the Open Dialogue approach, we also feel that the approach will benefit from additional research that can inform and further develop practice. Collectively our prior research has sought to consider how Open Dialogue is applied and practiced within different organizational settings (e.g., Buus et al. 2019; Buus and McCloughen 2022; Buus et al. 2021, 2022; Dawson et al. 2021; Ong and Buus 2021; Ong et al. 2023) with an overall aim of developing a more detailed, refined, and considered conceptualization of the approach. This study is accompanied by a second study (McCloughen et al. under review) focusing on the experiences of teachers in Open Dialogue meetings.

### 2.5 Ethics

The study was approved by the University of Sydney human research ethics committee and all participants provided written informed consent before data collection commenced.

### 2.6 Analysis

#### 2.6.1 Overview: From Reputation to Responsibility

**Figure 1:**
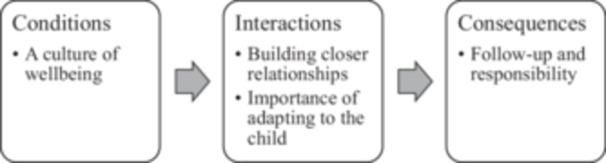
Themes arranged according to conditions, interactions, and consequences of the open dialogue meeting.

Our analysis constructed four themes: a culture of wellbeing, building closer relationships, the importance of adapting to the child, and follow‐up and responsibility. On reflection, we noticed that these themes could be organized into a chronological sequence of *conditions* occurring before the Open Dialogue meeting, the *interactions* within the Open Dialogue meeting itself, and the *consequences* arising from the meeting (Corbin and Strauss 2008) as depicted in Figure 1.

For the conditions before the Open Dialogue meeting, the school had a pre‐existing reputation with parents in the community that it offered something different from other schools, namely a focus on supporting students' wellbeing and mental health. This is captured in the theme of “a culture of wellbeing.” This culture of wellbeing set up the contextual conditions before the meeting and influenced the interactions in the school meeting itself and the consequences following it. The interactions during the meeting are represented by two themes. First, the meetings contributed to “building closer relationships” between the school staff and the students and parents respectively. Parents reported that they and their children got to know the school staff much better and knew who to contact if needed in the future. Second, the school meetings exhibited “the importance of adapting to the child.” This theme represents the efforts that adults made to help children feel comfortable in the meeting, focusing on eliciting the child's perspective, and developing ways to support them in the classroom. This theme also represents how parents were knowledgeable about the needs of their child and informed the school about ways to better support the individual needs of their children. The consequences following the meeting are represented by the theme “follow‐up and responsibility.” For this theme, parents expected a certain level of follow‐up in the form of changes to the way that teachers interacted with their child in class and through regular communication from the school back to parents.

In summary, by positioning themselves as focussing on student wellbeing and by eliciting feedback from parents and students at Open Dialogue meetings, the school promoted an open and collaborative environment, closer relationships, and a focus on the child. But some parents also held the school responsible for providing additional or altered supports for their children. Parents expected something more than what was provided within the meetings alone, which they saw as ultimately needing to produce tangible outcomes in the form of changed educational practices and consistent support for their children. This was an unexpected finding as the Open Dialogue literature focuses on the meeting itself and the promotion of collaboration within it, with little consideration about what happens before or after a meeting.

### 2.7 Conditions: Before the Meeting

A culture of wellbeing. Parents valued how the school operated and had chosen to send their children to this school due to its reputation for focusing on students' mental health and wellbeing. Parents noted that their children liked the school, were happier, and felt like they fitted in and did not stand out like in other mainstream schools. As their children attended school for a large part of each day, it was important for parents to know that they were being appropriately cared for, supported, and valued in addition to receiving an education. In Extract 1, two parents are discussing the positive points of sending their children to this school and how the school's focus on wellbeing had made a difference to their children's mental health.

Extract 1 FG2

***Participant 9:*** Had our child been at a mainstream public school he probably wouldn't have the same level of good mental health that he has at the moment because there's just lots of things that are part of the school culture [at this school] that help him feel like this is a real place of belonging for him, which he probably wouldn't have got anywhere else.

***Participant 8:*** Yeah and you know it's been said to me by a couple of parents here at the school, “[this school] exists, because there are parents like us who want not just a standard public school education, that we want more, we want more care, we want more responsibility for our children, we're not paying fees for nothing,” because I'd be happy to go on a holiday every year instead of pay school fees, but I'm paying school fees because I believe that [this school] is doing more, because their heart's in the right place.

These parents want more than what is provided at other schools and are willing to make financial sacrifices to pay for a school that offers more care and responsibility for children, which they notice has had positive effects for their children. Parents see themselves as active contributors to the school because without them it would not be funded and may not exist. The school and parents thus have a symbiotic relationship centered around mutual goals of promoting a caring and wellbeing‐centered school environment.

The school's culture of wellbeing was demonstrated in several other ways. First, the school often initiated meetings. Parents appreciated this as it showed the school's concern and acknowledgment that there were difficulties that needed to be addressed, and it showed that the school values parents' and children's perspectives. Second, parents could also initiate meetings, and the school was welcoming and responsive to these initiations. However, at times parents could feel frustrated as it can take a while to organize a meeting and to get all the necessary people, such as close family members, teachers, and external professionals to attend at the same time. Sometimes it felt like the parents had to “push” to get the meeting arranged and that the school was a little slow or reluctant to have the meeting.

Parents also reported some uncertainty about the meetings. While the reason for the meeting was often clear (e.g., due to some problems about attendance, mental health, learning support) it was not clear to parents if the meeting format was any different from other types of meetings with the school or if the meeting was routinely offered for all new students to the school. Some parents also had worries about attending a meeting as the school is a place of authority and parents could feel anxious about whether the school will be supportive or if the family will be subject to scrutiny and consequences.

### 2.8 Interaction: The Meetings

Building closer relationships. One of the primary outcomes of the Open Dialogue meeting reported by parents was a building and strengthening of relationships. These relationships were of two types: the relationship between the teacher and the student, and the relationship between school staff and parents. Parents stated that students initially felt uncomfortable in the meeting, but that school staff running the meetings were sensitive to these feelings and put a lot of effort into making the child feel comfortable and able to contribute to the meeting. This helped build the relationship between the child and the school staff by showing that the school was interested, trustworthy, and willing to help. Parents stated that after the meetings, students had a closer relationship with teachers and seemed more comfortable to approach teachers in future if needed. In Extract 2, a mother describes her son's experience in building a closer relationship with his teacher through the Open Dialogue meeting. The meeting was initiated by the teacher who noticed that the student often looked tired and wanted time outside the classroom.

Extract 2 FG1

***Participant 4:*** …it was a really good experience including [child's name] I could see that [the child thought] “oh finally somebody is listening to what I like or what I need in the class.” So, it was really positive. And I think he already felt good with his teacher, he really likes his teacher, but I think it helped him to build that relationship even further. I think it opened up more of a dialogue between him and the teacher. So, he feels like: “oh, if I need a break, I can go and speak to him straight away, because he knows exactly why I need a break.”

The mother describes how the Open Dialogue meeting helped her son to feel that his needs were finally being listened to, which had not happened in his previous meetings at other schools (data not shown). The Open Dialogue meetings thus facilitated a sharing of information and increased teachers' understanding of their students' experiences. This closer relationship was perceived to continue outside of the meeting and into interactions in the classroom.

Parents also developed closer relationships with school staff during meetings. Through the Open Dialogue meetings, parents learned who were working with their children and who to contact in future if required. The meetings were characterized by a mutual respect by all those present and a feeling that they were working together, even when there was disagreement about plans. Parents felt supported by the school who they felt could now better understand the difficulties that parents were facing with their children. This was especially so when the problems were complex and without easy solutions, such as when parents were struggling to get their children to attend school. There was a feeling of working together and being supported by the school even though progress could be slow, incremental, and long term. In Extract 3, a mother describes a feeling of support from having multiple staff members present in the meeting.

Extract 3 FG1

***Participant 1:*** Yeah, I was just so impressed with how many people were there. At the previous school it was usually two people. And there was probably four or five people in the meeting [from this school]. So, I thought that was good. The fact that there was not just the teacher, but the wellbeing officer and the team, it felt like a team supporting me.

This mother describes how having multiple people present in the meeting was a positive aspect. However, it was not only the presence of multiple staff members that provided a feeling of support as some parents reported meetings with only two staff members present. The significant element was the way that the meetings were conducted. In Extract 4, a father describes the feeling of support that arose from the mutual respect in a meeting.

Extract 4 FG3

***Participant 14:*** People are very respectful to one another, like we're all from different walks of life, different upbringings, different belief systems. And, I have no doubt when I come to these meetings, I can talk about how I feel, it may even be in disagreement with the school's policy, but I have never doubted I could come in and be honest and open about what I think is needed, which I'm not convinced you would always get at a school. So that's been very, very good for me, personally, probably more for me personally than the children.

This father comments on the mutual respect that was present in the meetings. He felt able to speak openly even when he disagreed with the school, which had a positive effect on him personally. The meetings were thus not only benefitting the child but also supporting parents.

The importance of adapting to the child. According to the parents, children held a privileged but vulnerable position in the Open Dialogue meetings. The meeting focussed on the needs of the children and their input into the meeting was actively sought and valued. But children were also seen as vulnerable and needing care and protection and adults adapted to the child's preferences so that they could actively participate in the meeting and feel supported. This is displayed in Extract 5, where a mother is talking about the vulnerable position of a child within Open Dialogue meetings.

Extract 5 FG2

***Participant 9:*** We need to specifically really protect children [in Open Dialogue meetings], right? Because as an adult, you can come out of it [the meeting] and process your own vulnerabilities in that moment. But when you're a young child, I think that sometimes that consideration isn't taken as much, because it's like, “oh, we're giving you the chance to consult on what's happening for you,” without thinking there does need to be some protections put in place in how we handle that. So, I do think that acknowledgment of how vulnerable people are in that space is really important.

In this extract, children are represented as vulnerable within the Open Dialogue meeting and do not have the ability to mentally process this vulnerability. This notion of vulnerability is mentioned and accepted by other participants but is not clearly defined. In this extract, *vulnerability* seems to refer to the child's emotional experience of the meeting and whether they can understand and manage these emotions. This parent suggests that the Open Dialogue meeting, and asking a child what is happening for them, are potential sources of harm. Consequently, adults need to have an awareness of a child's emotional state and take measures to protect children within Open Dialogue meetings. This vulnerability is also represented in the attempts to make the child feel comfortable in the meetings so that they were able to contribute and inform the adults about their experiences. In Extract 6, a mother is talking about her son reacting to too many questions by shutting down or getting angry and “blowing up” and the importance of adults being careful in how they react to him.

Extract 6 FG3

***Participant 13:*** When my son was ready to shut down or it was getting too much, we all learnt, and we probably as parents learnt earlier than the teachers, not to push, not keep asking questions… maybe a little bit early they pushed and that's when he would blow up but now, it's less likely, because they'll “we'll just leave him, calm, cool himself.” And then you know, for him, we know that 20 min later, if you leave, let it go, he'll be back and “oh, I'm really sorry I was like that” and move on. He's very switched. So, getting to know that about him, getting to learn about that particular person and that's happened fairly quickly, I think, in the [Open Dialogue] meetings.

This extract also demonstrates the flow of information in meetings. Generally, this flow of information was from the child, parent, and external professionals, who have a more knowing position about the child's behavior patterns, to the school who were perceived as being in a less knowledgeable position. In this extract, the Open Dialogue meetings facilitated this process of knowledge transfer to the school more quickly. Parents found it disappointing if not all relevant people were able to attend the Open Dialogue meeting because important information could not be transferred first‐hand if not all teachers were present to hear it (data not shown). This created a feeling of uncertainty whether the necessary changes would be implemented consistently across school staff.

### 2.9 Consequences: After the Meeting

Follow‐up and responsibility. The Open Dialogue meetings often did not immediately fix the problems and there was a need for follow‐up meetings. Parents felt that this was a normal part of the process due to the complexity of their situations and the child's difficulties. Some parents however, felt that the school could do more and wanted more specific outcomes and follow‐up after the meeting. Extract 7 demonstrates some parents' desires for regular communication as well as frustration about the school not following through on the plans that they thought were agreed upon.

Extract 7 FG2

***Participant 7:*** I definitely feel that I would like follow‐up to be consistent and ongoing so that we know what's happening and so that when we can't get all of the people working with our child together, we can at least communicate what's working and what's not, so that everyone can give him consistent support. Because that's really important. And it does often happen that you have a really good conversation, but then you're not quite sure what happens unless you really push to kind of say “I need to know what's happening next” and we [parents] are driving that.

***Participant 10:*** (…) I'm clear on what I think they're going to do but the next couple of weeks of talking to my son about what's happening in class doesn't always sound like something, what I thought was agreed at the meeting has actually been implemented in a consistent way across all the teachers. I know they all heard it because I was there when we spoke about it. But yeah, the follow through doesn't always happen. And these, these [Open Dialogue] meetings need to be more than just a way of us all feeling good. It's supposed to be more than the school giving you a hug. It's got to be a practical change in the classroom.

These parents felt that the Open Dialogue meetings offered valuable and supportive conversations, like the school “giving you a hug.” However, they were disappointed with the actual follow‐up after the meeting. Parents felt that the school's commitment to follow‐up was not always clear and that they had to “push” for the school to tell them what was happening next or to commit to a concrete plan. If a plan had been agreed upon in the meeting, parents expected regular communication from the school on how the plan was progressing and expected teachers to consistently implement the changes that were discussed in the meeting. For these parents, the Open Dialogue meeting was not viewed only as an intervention or a therapeutic process for the family. Instead, these parents viewed Open Dialogue meetings as the beginning of a change process characterized by a sharing of information that could later be implemented by teachers in the classroom.

## 3 Discussion

This study reports one of the first applications of Open Dialogue in a school setting, that is initiated and hosted by school staff rather than external mental health services. Our findings emphasize not only the importance of the therapeutic meeting itself, but also activities before the meetings, illustrated by the school's reputation in the community, as well as after the meeting in what follow up is provided. We discuss the implications of this both for practicing clinicians and for theorizing in the Open Dialogue approach.

Some parents in this study reported a dramatic difference in their children resulting from Open Dialogue meetings compared to the types of meetings they had experienced in other schools. While the beneficial outcomes of Open Dialogue meetings in schools needs further research and validation, schools could consider adopting Open Dialogue or some similar collaborative approach to complement their existing forms of meetings with students and parents. This may be especially useful when there are families experiencing complex problems as it encourages the discussion of multiple perspectives and new ideas. As reported by parents in this study, Open Dialogue can save time by bringing together all the important people and services around a child to share information and make collaborative plans. Open Dialogue meetings may also be useful for new students and families to promote discussions about their needs, to establish connections with their supports at the school, and anticipate and mitigate future problems.

However, as a result of the meetings, some parents viewed the school as responsible for making changes to support the child and communicate the progress to parents. This was an unexpected finding that highlights gaps in the theorizing of Open Dialogue practice. The Open Dialogue literature pays little attention to factors outside of the meeting such as the social context and the individual situation of each family. Instead, emphasis is placed on responding sensitively and dialogically to each prior utterance that occurs within a meeting (Haarakangas et al. 2007; Olson et al. 2014; Ong and Buus 2021). This study demonstrates that Open Dialogue and any other therapeutic meetings do not occur in a vacuum and that contextual factors do matter for parents. Within individual and family therapy, engagement and the therapeutic relationship are the most important contributors to client improvements (Duncan et al. 2010). Our findings suggest that while the meetings play an important role in building relationships, there is also significant influence from the reputation of the school in promoting student wellbeing in the community. For family therapists, this means a good therapeutic relationship is not restricted only to therapeutic meetings, it is also influenced by a broader engagement with the community to build a service's and a provider's reputations.

There are also broader social contexts that Open Dialogue practitioners need to recognize and consider in their work with families. Social values are dynamic and vary across generations. Out late modern society's increased focus on protecting children from risks (Lupton 2013) promotes greater scrutiny and pressure on parents to adhere to current expectations of parenting (Lee et al. 2010). Social researchers have described a culture of *intensive parenting* where parents (usually mothers) are expected to be task and achievement‐focused and invest extensive time and resources to provide their children with certain types of toys, food, discipline, play opportunities, extracurricular activities, and educational support (Ennis 2014; Hays 1996; Smyth and Craig 2017; Suissa 2006). Intensive parenting thus extends beyond a parent's monitoring for deficits in mental health and wellbeing, to promoting and advancing a child's achievement and potential beyond the average. Intensive parenting ideals have become widely accepted across social classes and cultures with parents generally accepting that intensive parenting is what good parents do to mitigate future risks for their children (Ishizuka 2019; Klimor Maman et al. 2024; Lee 2023; Smyth and Craig 2017). In addition, by fully accepting and investing in the ideals of intensive parenting, a parent can experience a positive transformation and self‐assertion of their identity and individuality (Paltineau 2014).

Intensive parenting can also have negative effects on parents. Parents who do not meet the standards expected of intensive parenting, often low‐income parents disadvantaged by less time and money (Romagnoli and Wall 2012), are seen as needing additional support and intervention. Parents' activities may thus be heavily scrutinized with schools, various government and nongovernment services, and even other parents potentially monitoring and reporting risks to children to protective services (Evans et al. 2023; Keast 2021; Lee et al. 2010; Shirani et al. 2012).

Therapists working with families of school‐aged children need to consider such social conditions and how intensive parenting may set up expectations that can leave parents feeling inadequate, guilty, under pressure, and stigmatized if they do not meet the expected standards (Romagnoli and Wall 2012). To maintain a positive parental identity, parents can reject the advice from therapists or intensive parenting programs and focus on their own parenting values (Romagnoli and Wall 2012; Shirani et al. 2012) or modify their values and expectations (Lee 2023). Similar to the findings of this study, parents can claim a position as expert knowers of their children by advocating greater service involvement or more appropriate service provision (Andrews 2014), or by speaking for their child and making decisions in the child's best interests (Neumann et al. 2021).

An Open Dialogue approach may provide a means of counteracting the potential blame and demands placed on parents, by valuing multiple equal perspectives, so parents can be invited to participate without having to defend their expertise or identity. However, parents who are not initially familiar with this approach may expect the usual types of school meetings they have experienced in the past and be primed to present a defensive and expert position. By maintaining a position of expertise on their child and directing the school to act differently, parents can guard against scrutiny and stigma, maintain a positive parental identity, and resist criticism from government services (Andrews 2014). Open Dialogue and family therapy practitioners thus need to actively engage with the broader social context such as the beliefs and attitudes of a culture (Bronfenbrenner 1977) rather than focusing only on the microsystems of a family and school or the dialogical conversation. In a school setting, this involves work on understanding parents' expectations of school meetings, as well as an awareness of the potential scrutiny and sanction that a school meeting may inadvertently impose. Therapists also need to set up clear expectations for parents on the collaborative intentions of an Open Dialogue meeting while remaining aware that firmly established social expectations may not be easily countered or resisted. A therapist's intention to collaborate with families is not enough. Collaboration and engagement are only brought into reality through purposive and continuous effort and refinement though an ongoing series of interactions.

We propose that for any school meeting with parents it is important to explicitly set clear expectations for the meeting and to understand each parent's expectations. If following a collaborative approach such as Open Dialogue, this discussion would occur at the beginning of the meeting when everyone is present, which may avoid potential misunderstandings and offset any contrary parental expectations. These discussions could include not only the purpose of the meeting and the potential outcomes, but also the unique aims of Open Dialogue where the staff will actively seek out the different perspectives of all participants. Similarly, an Open Dialogue approach tends to leave meetings open‐ended as there is an assumption that dialogues are “unfinalizable” and continuing (Seikkula and Trimble 2005; Shotter 1996). Our study suggests that parents may perceive their information‐giving to the school as requiring some follow‐up and action. It may therefore be necessary to end Open Dialogue meetings with a more focused discussion on specific actions to be taken after meetings to avoid such misunderstandings. This could be supported by clearly documenting the actions that will occur after the meeting and who is responsible for following them through. A copy can then be given to all participants so parents are clear on the plans, what follow‐up communication they can expect, and who to contact if there is a problem.

Therapists may also have to maintain flexibility in their use of Open Dialogue meetings to be appropriately responsive to families. For example, the parents in this study noted the potential vulnerability of students in such a meeting. Therapists should therefore be aware that an open conversation can be confronting for some students and will need strategies to manage any problems such as student distress or anger. A discussion with parents before the meeting may help identify such risks. Therapists also need to be flexible in adapting to the emerging needs and preferences presented by students and parents. For example, parents may be seeking a clear decision or outcome from the meeting. In this case, the school counseling staff would need to consider whether to continue with an Open Dialogue approach or adapt the meeting to become more problem and task‐oriented.

Another issue of practical importance is the distribution of expertise. In contrast to traditional health care approaches where clinicians have expert knowledge about assessment, diagnosis, and treatment, Open Dialogue alters this arrangement of expertise, where the client and family are instead viewed as having expert knowledge about their situation and the therapist has expertise around facilitating the conversation (Anderson and Goolishian 1988, 1992; Haarakangas et al. 2007; Seikkula and Arnkil 2006). This has been demonstrated in prior research where clinicians downgrade their own knowledge and defer to clients but still direct the conversation in subtle ways (Ong et al. 2023). By refraining from providing solutions to clients' problems, clinicians transfer responsibility for change to clients rather than taking it up themselves (Buus and McCloughen 2022). Similarly, in this study, the school staff took a less knowledgeable position about the child and invited input from parents and students. Consequently, parents were often positioned as experts on their child, acquired through their past experiences with their child as well as prior contact with external professionals. The elevation of parental expertise may therefore promote more collaborative relationships and feelings of being important and valued by clinicians. But, by inviting open discussions and subsequently being informed by parents, the school staff became responsible for following up on any suggestions that arose from those meetings. For family therapists, this means that developing collaboration with clients also involves giving up some power and authority. Traditional hierarchies of therapist authority, place greater weight on therapists' formulations and directives. More equal collaborative relationships, opens possibilities for therapists and their models of working to be challenged by clients. Therefore, a commitment to collaboration, also means a commitment to uncertainty and a questioning of accepted professional knowledges.

### 3.1 Limitations

This study was conducted in a unique setting of a small independent school within a village community and a staff group focused on student support and wellbeing. In such a setting the school can take a more prominent role in the community encouraging parental engagement and involvement rather than a site only for education. Similarly, the school had been trialing various types of collaborative meeting styles before introducing Open Dialogue. Consequently, the leadership team within the school had been cultivating a wellbeing culture for many years. This means that the introduction of Open Dialogue may not have been such a leap for teachers or parents within this community. In schools with a different culture, different leadership priorities, greater separation of roles, and less involvement of teachers in student wellbeing, the introduction of Open Dialogue would be more difficult with less acceptance and different parental experiences. This setting thus lends itself to self‐selection of a particular type of parent and student group that is likely not representative of other schools. This self‐selection may represent a range of reasons such as availability of a group of parents who are particularly interested and active in engaging with the school over their children's education. Consequently, this may have contributed to the parents' appreciation of being invited into Open Dialogue meetings, as well as their expectations of more active follow‐up and communication from the school. Larger schools in more urban areas with relatively less parental involvement may have very different experiences of such collaboratively styled meetings. In addition, this study did not seek specific demographic information from families such as structure, culture, nor ethnicity and these factors may have also influenced their responses to the Open Dialogue meetings.

Focus group research can gather multiple perspectives in a short time frame however, it may also lead to agreement within a group and less diversity of perspectives. For this study, we elected to use focus groups due to their alignment with the Open Dialogue approach, the potential for cohesion between parents with similar experiences, and the possibility of new ideas emerging through their discussions.

The level of fidelity in the implementation of Open Dialogue is difficult to determine as it is centered around the ideas of flexibility and responsiveness. Consequently, Open Dialogue does not lend itself well to the approaches of implementation science that require manualised and quantitatively operationalizable principles that can be reliably measured (Waters et al. 2021). While there have been a number of attempts recently to develop fidelity principles for Open Dialogue (e.g., Lotmore et al. 2023; Olson et al. 2014; Ziedonis et al. 2020), these are yet to be validated and continue to utilize concepts that are difficult to reliably operationalize (Freeman et al. 2019; Waters et al. 2021). Therefore, in this study, the extent to which Open Dialogue principles were followed is difficult to determine outside of the training, intention, commitment, and self‐regulation between staff.

Our research did not analyze differences in the presenting problems of students nor the number of meetings held between parents and staff. It is possible that different types of problems and diagnoses would result in different types of conversations in school meetings. For example, relatively complex problems such as school refusal do not have simple solutions and are therefore often accompanied by multiple meetings. This could potentially create different types of relationships as parents and staff can both be struggling to find solutions creating a greater sense of involvement and togetherness.

### 3.2 Future Research

Parental expectations both before and after a meeting were significant findings. Future research could focus more specifically on what parents' hopes, expectations, and concerns are before meetings. This could provide therapists with clearer ideas on what internal and external pressures families are experiencing. Similarly, as there appears to be differences in how parents and the school staff perceived the follow up actions after a meeting, future research could compare post‐meeting interviews or questionnaires on what parents and staff expected would happen after meetings. These could also be compared against recordings of the meetings to identify potential sites of conversational problems in communicating follow up. This would have practical utility to identify problematic communication practices that can inform practice.

Future research may consider the longer‐term use of Open Dialogue in a school setting and examine if the adoption of the approach continues to lead to similar outcomes and accountabilities when parents are more familiar with the approach. Similarly, different types of presenting problems and number of meetings may create different levels of engagement and feelings of relative responsibility that are not clear from this study. The perspectives of students themselves is an important aspect that is missing from the current research. We found that students were presented as both central to a meeting but also vulnerable with teachers and parents sensitive to maximizing a student's participation while also being sensitive to their emotional vulnerabilities. Future research could provide a more comprehensive view of Open Dialogue meetings through focusing on the perspectives and experiences of students from a range of age groups.

## 4 Conclusion

An Open Dialogue approach to school meetings presents an alternative to goal‐directed planning and outcome achievement by focusing on the voicing of multiple perspectives and tolerating uncertainty in problem‐definition and goal planning. Parents reported that an Open Dialogue meeting contributed to a closer relationship with the school. However, some parents considered the school to be responsible for making changes after the meeting. Our findings suggest that Open Dialogue meetings actively seek the input of parents thus positioning them as experts on their children with useful information to share with the school staff. This creates opportunities for a more collaborative decision‐making process and closer supportive relationships between students, teachers, and parents but also decreases the school's control over knowledge and determining how plans are decided in a meeting. We suggest that when adopting a collaborative approach such as Open Dialogue, family therapy clinicians need to be mindful of broader social contexts and expectations, which influence how people may experience and participate in a meeting, rather than focusing solely on the micro‐culture of the family and the dialogical interaction. This may involve taking time to meet with parents about their expectations of the school and the meeting, setting clear expectations with parents before a meeting on how an Open Dialogue meeting is conducted, and being clearer on what follow up actions are to occur, preferably in writing. In addition, a school's position and engagement in a community can have potential impacts on parental engagement even before meetings begin. So, while a commitment to Open Dialogue creates a supportive and collaborative environment for parents, therapists must also be aware of the expectations that parents bring to a meeting.
